# Safety considerations in the bioanalytical laboratories handling specimens from coronavirus disease 2019 patients

**DOI:** 10.4155/bio-2020-0185

**Published:** 2020-08-21

**Authors:** Zhuo Chen, Timothy W Sikorski

**Affiliations:** ^1^Bioanalysis, Immunogenicity & Biomarkers, In-vitro/In-vivo Translation, R&D Research, GlaxoSmithKline Pharmaceuticals, 1250 South Collegeville Rd, Collegeville, PA 19426, USA

**Keywords:** coronavirus inactivation, COVID-19, SARS-CoV-2, viral load in matrices

## Background

The outbreak of coronavirus disease 2019 (COVID-19), which is caused by severe acute respiratory syndrome coronavirus 2 (SARS-CoV-2), has been declared as a public health emergency of international concern and then reclassified as a pandemic by the WHO [1,2]. Similar to severe acute respiratory syndrome coronavirus (SARS-CoV) and Middle East respiratory syndrome coronavirus (MERS-CoV), SARS-CoV-2 is a β-coronavirus causing mainly pneumonia with high mortality rates [3,4]. In this global crisis, numerous healthcare professionals and laboratory workers still work daily to combat the COVID-19 pandemic. In particular, personnel in bioanalytical laboratories who handle clinical samples, now need to take new special precautions in the event these samples are SARS-CoV-2 positive. Therefore, the purpose of this commentary is to summarize the viral load and infectious risk of SARS-CoV-2 in different biological matrices based on recent reports. The effectiveness of the published viral inactivation protocols before sample processing and their potential interference with bioanalytical assays are also reviewed. Here, we also discuss appropriate laboratory operations to minimize exposure risks. We hope that the bioanalytical community can benefit from this article by understanding the risk of SARS-CoV-2 transmission when handling clinical specimens from COVID-19 patients and improving laboratory biosafety practices.

## SARS-CoV-2 viral load in biological matrices

A main safety concern for bioanalytical laboratories handling COVID-19 patient samples is the SARS-CoV-2 viral load in biological matrices. Some recent studies have reported SARS-CoV-2-positive detection rates in patient specimens. Most of these studies employed reverse transcription-polymerase chain reaction to detect viral RNA for the diagnosis of SARS-CoV-2 infection [5,6]. Although the statistical data vary somewhat due to the difference in sample size and patient’s disease severity, the observations were generally consistent. Since the virus primarily targets the respiratory system, positive rate of SARS-CoV-2 was the highest in respiratory matrices as expected, with higher viral loads detected in lower respiratory specimens (e.g., bronchoalveolar lavage fluid and sputum) than upper respiratory specimens (e.g., nasal swab and throat swab). Wang *et al.* collected 1070 specimens from 205 COVID-19 patients with the mean age of 44 years (range from 5 to 67 years and 68% were male) [7]. Most patients showed symptoms of fever, dry cough and fatigue; 19% of patients had severe illness. SARS-CoV-2 was positively detected in 14 of 15 (93%) bronchoalveolar lavage fluid, 75 of 104 (72%) sputum, five of 8 (63%) nasal swabs, 126 of 398 (32%) throat swabs, 44 of 153 (29%) feces, three of 307 (1%) blood and none of 72 (0%) urine [7]. Yu *et al.* analyzed 323 specimens from 76 COVID-19 patients with the median age of 40 years (range from 6 months to 92 years and 50% were male) [5]. The major symptoms were fever (88.2%) and cough (69.7%); 77.6% of patients were the mild type, while 22.4% were the severe type. The positive rates detected from patients were 77 of 116 (66%) in sputum, nine of 55 (16%) in nasal swabs, 50 of 134 (37%) in throat swabs, none of 4 (0%) in blood and none of 14 (0%) in urine [5]. Another study specifically focused on viral load in clinical samples of critically ill patients with COVID-19 requiring Intensive Care Unit admission. Among these 16 patients with the median age of 59.5 years (range from 26 to 79 years and 81% were male), SARS-CoV-2 was detected in 16 of 16 (100%) sputum, 13 of 16 (81%) nasal swabs, 10 of 16 (63%) throat swabs, 11 of 16 (69%) feces, one of 16 (6%) blood and one of 16 (6%) urine [6].

Since blood specimen is one of the most commonly analyzed sample types, viral load of SARS-CoV-2 in blood is a key issue for laboratory biosafety. Besides the above three studies, other recently published papers also demonstrated that the detection rates of SARS-CoV-2 RNA in blood from COVID-19 patients were generally low as: five of 48 (10%) [1], two of 9 (22%) [2] and six of 41 (15%) [3]. These results were consistent with the previous studies on other coronaviruses, which showed a much lower viral load in the nonrespiratory specimens including blood and urine [4,8]. Direct contact and airborne transmission are considered the major routes of spread of coronaviruses. Moreover, detection of viral RNA in the matrix does not mean that the RNA retains its original length or is part of a live infectious virus. So far, no research has observed infective SARS-CoV-2 particles in blood [9]. Finally, a recent study compared the detection of SARS-CoV-2 RNA in serum of 48 patients with varying severity [1]. Although all throat swab specimens from these patients were tested positive, only five showed positive detection in serum and all came from critically ill patients (two of whom died of the disease). No viral RNA was detected from serum in the mild or severe cohort, which may represent the majority of COVID-19 patient samples. Therefore, according to current knowledge, the risk of processing blood specimens from COVID-19 patients is not considered to be high. Nevertheless, there is still much work to be done in understanding the infectivity of blood derived samples from COVID-19 patients, and at this time any risk of exposure to laboratorians should be mitigated, as described below.

## Protocols to inactivate SARS-CoV-2

Despite the relatively low SARS-CoV-2 viral load detected in the blood and urine of COVID-19 patients, scientists have yet to draw conclusions about the infectivity of these specimens. The Centers for Disease Control and Prevention (CDC) has issued the interim laboratory biosafety guidelines for handling COVID-19 specimens, which recommends virus inactivation prior to sample processing to reduce the risk of infection [10]. Coronaviruses are enveloped ssRNA viruses that are usually thermolabile and unstable under acidic or alkaline conditions. However, they are more stable at 4°C and can remain infectious after 25 cycles of freezing and thawing [4].

At present, most reported protocols for inactivating SARS-CoV-2 are based on the inactivation methods for SARS-CoV and MERS-CoV. They are generally classified into two categories: heating protocols and chemical protocols. Wang *et al.* compared the infectious titer of SARS-CoV-2 after heating the samples at different temperatures for different periods [11]. They found that heating the specimens (human serum or sputum) at 56°C for 30 min efficiently inactivated any infectious SARS-CoV-2 while preserving viral RNA. Unlike some other thermolabile coronaviruses, SARS-CoV-2 was observed to be stable at 37°C for at least 24 h. This finding may partially explain why COVID-19 outbreaks can occur in some tropical countries. Another study also showed that heating at 56°C for 30 min was sufficient to inactivate the blood specimens with low viral load [12]. Nevertheless, only the protocol of heating at 92°C for 15 min completely inactivated SARS-CoV-2 in respiratory specimens with much higher viral load. Since the detectable viral RNA was also drastically reduced under this condition, this protocol is not compatible with subsequent reverse transcription-polymerase chain reaction detection (if required). Among the various chemical protocols, lysis buffer with detergent is the most commonly employed. Pastorino *et al.* found that 1–10% sodium dodecyl sulfate buffer or 30–50% guanidine hydrochloride/1–10% *t*-octylphenoxypolyethoxyethanol (Triton X-100) effectively inactivated SARS-CoV-2 with viral load as high as 10^6^ TCID_50_/ml [12]. Some protocols utilized reducing reagents to denature viral proteins by reducing disulfide bridges. For instance, a shelf-stable reducing agent tris(2-carboxyethyl)phosphine was used for SARS-CoV-2 inactivation when mixed with the divalent cation chelator EDTA and heated at 95°C for a brief period (5 min) [13]. This protocol rapidly denatures proteins while protecting the viral RNA from the degradation by endogenous RNases. The addition of EDTA sequesters any divalent cations that are released from denatured proteins and that are required for RNase activity. Some photochemical protocols are also applicable, such as treatment with riboflavin/ultraviolet light [14] or methylene blue/visible light [15]. However, the photochemical protocol may require specialized equipment not available in all laboratories.

It is worth noting that both heating and chemical protocols may denature the protein analyte of interest in a bioanalytical assay, resulting in measurement bias or false-negative results. For example, a paper demonstrated that heat inactivation of serum at 56°C for 30 min significantly interfered with the quantitation of antibodies to SARS-CoV-2 in a fluorescence immunochromatographic assay [16]. Of the 34 serum samples from COVID-19 patients, the IgG levels in 24 samples dropped by an average level of 49.54%. The IgM levels of all 34 samples decreased by an average level of 53.56%. Moreover, 44.12% of the IgM signals reduced below the cut-off value after heating, indicating that the false-negative results were caused by the heat inactivation. Therefore, the possibility of analyte denaturation and its impact on the accuracy of immunoassays or other bioanalytical techniques should be considered when introducing an inactivation protocol. Peptide-based LC–MS assay is compatible with protein denaturation and can therefore be considered as an alternative approach for the analysis of proteins from inactivated samples.

## Appropriate laboratory practices for handling specimens containing SARS-CoV-2

The latest guidelines by the National Institutes of Health (NIH) classified SARS-CoV and MERS-CoV as risk group 3 agents and other coronaviruses as risk group 2 agents [17]. This classification was released before the emergence of SARS-CoV-2, but the consensus is to classify SARS-CoV-2 as a risk group 3 agent. Based on this classification, the interim guidelines from both WHO [18] and CDC [10] recommend that nonpropagative laboratory work on clinical specimens from patients who are suspected or confirmed to be infected with COVID-19 should be conducted in the Biosafety Level 2 (BSL-2) laboratory. Biosafety Level 3 laboratory is required for propagative work (e.g., virus culture or neutralization assays) when handling materials with high concentration of live SARS-CoV-2 virus. Therefore, bioanalysis of COVID-19 patient samples, including both respiratory specimens and nonrespiratory specimens, should be performed in a BSL-2 laboratory [8]. In addition, the CDC guidelines recommended the site-specific and activity-specific biosafety risk assessments to determine whether additional biosafety precautions should be taken based on situational needs. Also, local regulations need to be complied, if certain countries or regions have unique or specific requirements for handling samples from COVID-19 patients.

A paper by Cossarizza *et al.* [9] described the procedures they followed for processing blood specimens from COVID-19 patients: the patient’s blood samples were handled in the BSL-2 laboratory supplied with a certified Class II Biosafety Cabinets (BSC), which was daily equipped with an internal waste (containing 0.5% bleach) to discard the contaminated biological materials. To reduce the risk of breakage, the samples were placed in two secondary containers that were opened inside BSC. In a Class II BSC, personnel are required to wear personal protective equipment including laboratory coat, eye protection, fit-tested N95 respirator or surgical mask and two pairs of disposable gloves. At the end of the operation, the outer layer of gloves is discarded into the internal waste of BSC. A social distancing of at least 1 m is strongly recommended between people inside the laboratory in case a laboratorian is asymptomatically infected and spreads the virus. van Doremalen *et al.* has reported that SARS-CoV-2 can remain infectious in aerosols for hours and on various surfaces up to days [19]. Therefore, laboratorians should take extra care and operate in a Class II BSC when performing experimental procedures that may generate aerosols or droplets (e.g., vortexing, mixing, plate washing, sonication and centrifugation). It is highly suggested to use safety buckets or sealed rotors for centrifuges. In addition, the surfaces of laboratory bench must be thoroughly cleaned with 62–71% ethanol or other disinfectants after use [18]. If a potential exposure to infectious materials happens, the line manager and Environment, Health, and Safety department should be immediately reported.

## Concluding remarks

The COVID-19 pandemic has had and will continue to have a huge impact on the entire world. Bioanalytical laboratories have had and will continue to play an important role in the development of treatments and vaccines against this deadly disease. Due to the high demand for rapid diagnosis and the historical speed and diversity of currently planned and ongoing clinical trials, large numbers of clinical specimens from COVID-19 patients need to be analyzed daily in bioanalytical laboratories around the world. This undoubtedly shows the commitment of bioanalytical scientists to improve the public health. At the same time, the laboratory management is responsible for providing a safe environment for those who are delivering high-quality results. Although SARS-CoV-2 is highly contagious, the risk of processing specimens containing SARS-CoV-2 is controllable and many reported protocols have proven to effectively inactivate viruses before sample processing. However, knowledge about SARS-CoV-2 is still limited. To ensure safety, staffs handling clinical specimens from COVID-19 patients should not underestimate the infectivity of samples and need to strictly follow the laboratory biosafety practices.
